# Engaging With Aging: A Qualitative Study of Age-Related Changes and Adaptations

**DOI:** 10.1093/geroni/igac054

**Published:** 2022-10-11

**Authors:** Shaoqing Ge, Kuan-Ching Wu, Hillary Frey, Maryam Saudagaran, Derick Welsh, Janet Primomo, Basia Belza

**Affiliations:** School of Nursing Department of Biobehavioral Nursing and Health Informatics, University of Washington, Seattle, WA, USA; School of Nursing Department of Biobehavioral Nursing and Health Informatics, University of Washington, Seattle, WA, USA; University of Washington Medical Center—Northwest, Seattle, Washington, USA; School of Nursing Department of Biobehavioral Nursing and Health Informatics, University of Washington, Seattle, WA, USA; Providence Regional Medical Center Everett, Everett, Washington, USA; School of Nursing and Healthcare Leadership, University of Washington Tacoma, Tacoma, Washington, USA; School of Nursing Department of Biobehavioral Nursing and Health Informatics, University of Washington, Seattle, WA, USA

**Keywords:** Independent living, Management, Nursing, Strategy

## Abstract

**Background and Objectives:**

In the context of global aging, there is a need to better understand how older adults adapt to their changing health status. Engaging with aging (EWA) is an emerging framework proposed by Carnevali, which provides a new lens to understand an active, conscious daily-living process of managing age-related changes (ARCs) taken on by older adults. Study aims were to (a) describe the ARCs experienced by community-dwelling older adults and (b) identify the strategies and resources used by older adults to accommodate the daily-living challenges caused by the associated ARCs.

**Research Design and Methods:**

We conducted semistructured interviews using a virtual card sort to gather qualitative data about ARCs and strategies to manage ARCs. Interviews were conducted virtually due to coronavirus disease 2019 (COVID-19) restrictions.

**Results:**

Participants included 19 females and 10 males. The mean age was 77.45 years old (range from 64 to 98). Sixteen ARCs (e.g., changes in hearing, changes in stability, changes in sleep, etc.) were mentioned by participants, and their corresponding adaptations were discussed. Participants linked their adaptations to their ARCs based on their changing capacities and needs. Examples of commonly used adaptations included, for example, conserving energy, utilizing tools or technology, and being more conscious before and while taking actions. The challenges caused by COVID-19 in implementing the adaptations were also discussed.

**Discussion and Implications:**

Findings from this study demonstrate how older adults explore, generate, and utilize adaptive behaviors to address their ARCS. This study substantiates the EWA framework by showing common patterns among older adults in linking ARCs with adaptations. Implications for clinicians include using EWA to help older adults identify personalized health solutions that fit their capacities. Researchers may use EWA to design and test interventions by considering the specific ARCs older adults encounter and the attitudes they hold towards the ARCs.

**Translational Significance:** This study examines the biological, behavioral, and environmental influences on older adults’ health and well-being by examining the experiences of age-related changes (ARCs) and associated adaptations from a nursing science perspective. This theory-driven qualitative study and in-depth analysis demonstrated that older adults developed individualized adaptations to fit their needs and capacities. Implications include that clinicians and researchers may use Engaging with Aging to develop interventions that better address older adults’ needs and help older adults better identify practical solutions for managing their ARCs.

The global population is aging rapidly. According to World Population Prospects, one in six people will be over the age of 65 by the year 2050 (United Nations, 2020). The U.S. Census Bureau (2017) projects that the number of older adults aged 65 years and above in the United States will nearly double from 52 million in 2018 to 95 million by 2060; their share of the total population will rise from 16% to 23% (U.S. Census Bureau, 2017). This requires that policymakers, researchers, and clinicians pay close attention to the health and well-being of older adults.

As a natural process of aging, older adults may experience diseases and age-related changes (ARCs). Age-related changes are defined as normal, progressive maturational developments accompanied by physical and cognitive changes associated with aging (Carnevali et al., 2019; Diehl & Wahl, 2010). By successfully adapting to ARCs, older adults can optimize function and quality of life, as well as reduce morbidity and frailty (Ge, McConnell et al. 2021; Naik et al., 2021; Rutherford et al., 2018; Su et al., 2019). Understanding these ARCs and the related adaptations that older adults make or need to make from the perspective of older adults can inform health care providers, community partners, and policymakers to develop person-centered, targeted programs and services to better address older adults’ needs (Carnevali et al., 2019).

Engaging with aging (EWA) is an emerging conceptual framework proposed by Carnevali, who is a centenarian and retired nurse (Figure 1). EWA provides a new lens to understand the process of aging: it describes an active, conscious process of managing ARCs that is implemented by an older adult in daily life (Belza & Primomo, 2019; Carnevali et al., 2019). According to Carnevali, EWA is “a perspective, an attitude, a framework, and set of processes that older adults may proactively use to manage their daily living in the face of emerging and progressing maturational developments and the incidence of pathology” (Belza & Primomo, 2019; Carnevali et al., 2019). Essentially, the EWA framework aims to provide a holistic overview of the dynamic interactions between biological age-related changes, environmental factors, and behavioral adaptations of older adults, which can inform how nurses and other healthcare professionals provide services that better meet older adults’ needs (Ge, Belza et al. 2021; Su et al., 2019). Compared with other concepts related to “adaptation” in aging (e.g., healthy aging, successful aging, etc.), EWA is unique as it emphasizes older adults’ active physical and cognitive engagement with the aging process in which they seek to discover and test adaptive strategies by applying various resources to accommodate their changing capacities, including physical, cognitive, psychosocial status that influence their daily living (Flood, 2002; Hansen-Kyle, 2005). Therefore, EWA fills a prior research gap by providing a framework that focuses on the constant adaptations to ARCs that emerge over the trajectory of aging, and provides an innovative theoretical angle to view the process of aging by focusing on the daily-living environment and experience, which can produce cumulative impacts on older adults’ quality of life and health outcomes (Ge et al., 2017; Lyu & Wolinsky, 2017; Tang et al., 1999). However, the concept of EWA has not yet been researched.

**Figure 1.**
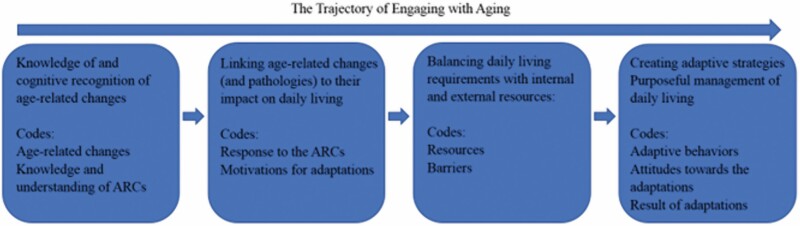
A graphic demonstration of the Conceptual Framework of Engaging with Aging and codes from the current study. Revised based on Carnevali et al. (2019). ARC = age-related change.

Therefore, the aims of this study were to use EWA as a guide to (a) describe the physical, cognitive, social, societal, and psychological ARCs experienced by community-dwelling older adults and (b) identify the strategies and resources used by older adults to accommodate the daily-living challenges caused by the associated ARCs. In this initial study of EWA, we are primarily focusing on older adults who are physically and mentally capable of managing their daily-living activities, as the conceptualization of EWA focuses on describing the positive behavioral patterns of this group of older adults.

## Method

A qualitative descriptive study was conducted to describe and understand the ARCs and adaptions experienced by older adults. We collected and analyzed data from semistructured qualitative interviews with older adults regarding their experiences with aging. Institutional Review Board (IRB) approval was received from the University of Washington (STUDY00010737).

### Sample and Setting

Individuals were recruited via convenience and snowball sampling from community partners (senior centers and a retirement association) in the greater Seattle and Puget Sound area in Washington. IRB-approved study flyers were posted in either the physical location of the facilities or distributed online via newsletters to members. The text in the flyer invited community members to participate in an interview about their daily-living experiences and ideas about aging. A $40 gift card was offered for participation. Participants were required to be age 60 or older, able to routinely perform daily activities (e.g., walking, eating, bathing) without help from others, and able to read and speak English. Interested participants contacted the investigator via telephone or email. To carry out the interviews during the coronavirus disease 2019 (COVID-19) pandemic, we pivoted from in-person to remote interviews via Zoom or telephone. Thus, participants were required to have access to the Internet or a phone.

### Data Collection

Eligible participants were scheduled for an interview over the online Zoom platform or telephone. Zoom platform training was offered to all participants prior to the interview. For the qualitative interview, participants received an email from the study team with instructions and links to (a) the consent form and (b) an initial list of ARCs for participants to review and consider whether they personally encountered or not. Interviews were conducted between November 2020 and February 2021. Each interview lasted 75–100 min with a participant and two study team members (based on the matching availability): one as the primary interviewer and the second for technology support. Interviews were audio recorded and uploaded to a secure online platform.

**Figure 2.**
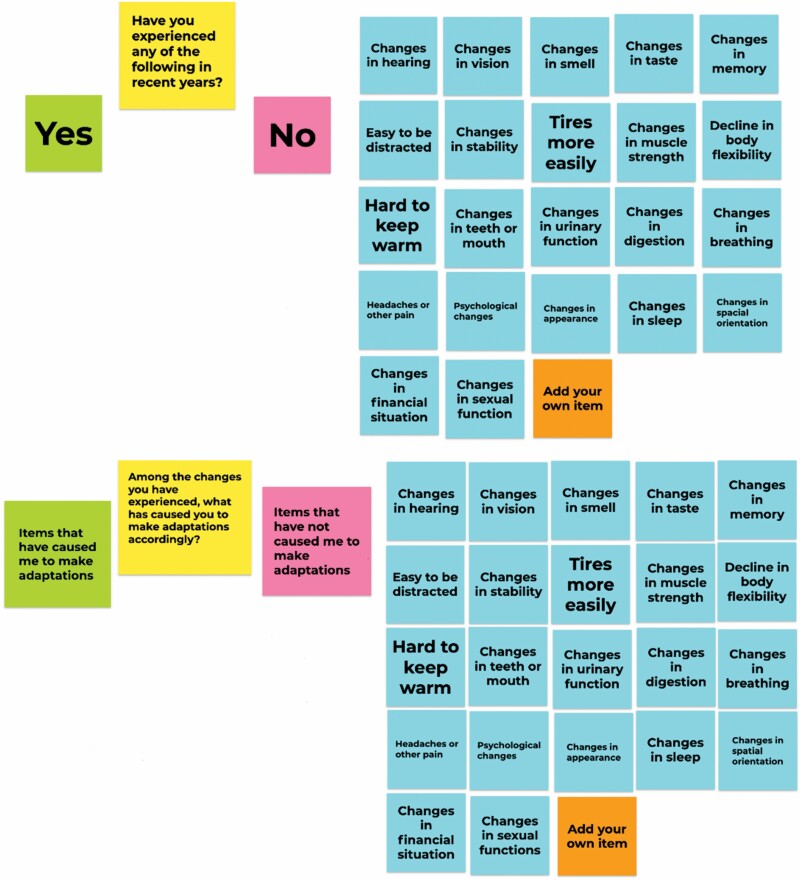
Google Jamboard containing post-it note cards used to assist the card sort during the interviews.

All team members received training in qualitative interview methods before conducting the interviews. Interviewers were undergraduate or PhD in Nursing Science, nursing students and faculty and had no previous relationships with the participants. Each interviewer used the same interview guide (Supplementary Material), which was developed and revised based on the results of a pilot study. The interview started with a virtual card sort activity, which is a qualitative elicitation technique to help participants assess their experiences and facilitate the classification of topics being explored (Mammen et al., 2016; Schwartz et al., 1998). Interviewers shared their computer screens and presented post-it note cards using a Google Jamboard (Figure 2). Each card included one common ARC based on Carnevali’s knowledge and experience in aging. Participants were asked to identify which ARCs had caused them to make adaptations in their everyday living, then to rank the top three changes that caused the most impact in daily living. The purpose of doing a card sort activity is not to calculate the frequencies of ARCs. Instead, the card sort was used to inspire participants to gain a deeper and broader reflection on their own experience with the ARCs. Next, participants were asked to describe their aging experience in their own words by sharing details about their experience with their top three ARCs and relevant adaptations. Participants were also asked about ARCs that had not caused them to make adaptions to their everyday living.

### Data Analysis

The interview recordings were transcribed by a professional transcriptionist. Transcripts were imported into the ATLAS.ti software to aid the qualitative analysis (ATLAS.ti 7 Windows). Because we used EWA as a conceptual framework to guide the analysis, we conducted an iterative coding process using a directed content analysis approach (Hickey & Kipping, 1996; Hsieh & Shannon, 2005). An initial codebook was developed using a priori themes from the EWA framework (Carnevali et al., 2019) and prior analysis of the EWA blog (Su et al., 2019) as well as line-by-line coding of the first three transcripts, and additional codes were added as they emerged from the data (Hsieh & Shannon, 2005). The codebook included definitions and examples of each code. All study team members coded the first three transcripts individually to compare coding styles and ensure trustworthiness between coders. The remaining transcripts were coded in couplets independently and then compared to ensure trustworthiness between the two coders. The data were continuously analyzed for emerging codes, categories (i.e., grouping of codes), contradictions, and overlaps.

Multiple strategies were used to establish qualitative rigor and trustworthiness (Elo et al., 2014). We held weekly meetings of the coding team to discuss the use of codes. Any discrepancies in coding that were not able to be resolved in the couplets were discussed in the weekly meetings. During the weekly meetings, we also discussed the use of codes, the coding, and definitions of codes. We compared our coding patterns to ensure that the interpretations made were consistent. We also engaged other team members who did not participate in coding so that they could review and critique our coding and interpretations. Quotes and rich descriptions were provided to contribute to the transferability. Using a qualitative approach allowed us to robustly describe the ARCs and adaptions experienced by older adults and examine the feasibility of using the EWA framework in health care educational and research settings.

## Results

### Participant Characteristics

All of the eligible participants who were invited agreed to participate in the study. Informed consent was obtained using REDCap from 30 older adults (20 females and 10 males). One interview recording became inaccessible due to a computer malfunction and could not be retrieved even after trying various methods. Therefore, 29 interviews were analyzed in the current study. The mean age was 77.45 years old (range from 64 to 98). Participants were mainly White (*n* = 27, 90%), with one African American (3%) and one Hispanic (3%) participant (Table 1).

**Table 1.** Sample Characteristics (*n* = 29)

Characteristics	Mean ± *SD*	Range	*n*	%
Age	77.45 ± 9.31	64–98		
64–69			5	17.2
70–79			14	48.4
80–89			5	17.2
90–98			5	17.2
Gender				
Male			10	34.5
Female			19	65.5
Years of education				
≤12 (HS diploma or less)			2	6.8
13–15 (some college)			3	10.3
16 (college degree)			7	24.1
17 (some postgraduate)			4	13.8
18 (masters, MA/MS)			11	37.9
≥19 (some doctorate)			2	6.9
Race				
White			27	93.1
African American			1	3.4
Hispanic			1	3.4
Marital status				
Never married			2	6.9
Married			15	51.7
Divorced			4	13.8
Widowed			8	27.6
Living situation				
Alone			12	41.4
Not alone			17	58.6

*Notes*: HS = high school; MA = Master of Arts; MS = Master of Science.

### Age-Related Changes

Sixteen ARCs were described during the interviews. Table 2 lists the ARCs and exemplars of capacity changes described by participants. Three ARCs—“changes in smell,” “changes in taste”, and “changes in spatial orientation” were available in the card sort but were not reported by any participant. “Changes in appearance” and “hard to keep warm” were chosen by a few participants but were not described in detail, suggesting that they were not of high concern to the participants.

**Table 2.** Age-Related Changes and Manifestations Among Participants

Age-Related Changes	Quotes
Physical related	
Tires more easily	“It’s just that you do not have as much strength as you used to. Although, I could still do everything. I just get tired” (P09).
Changes in hearing	“That’s so I can participate in things. I’m surrounded by people ... And children are difficult with hearing aids, because they have a high pitch and they often do not have very good enunciation. So the hearing is perhaps the highest in keeping me feeling that I participate in the world around me” (P02).
Changes in vision	“When they (changes in eyes) get really bad, no matter how many drops I put in them (eyes) I can’t read, and that disturbs me greatly. Like yesterday, I couldn’t read yesterday, but today I can read. It’s intermittent” (P17).
Changes in stability	“Hills and stairs are a challenge to stability because I’m on one foot part of the time. I’m walking up or down and flexibility plays in because when I’m off-balance, I try to recover my balance and my body is less flexible than it used to be” (P14).
Changes in muscle strength	“The tasks that I would think I could do easily have become harder. Just opening up a jar and finding that my hand strength is not the same for opening up that jar ... If both grandchildren want to jump up on me and I’m called upon to lift both of them, I notice that’s harder and harder. Part of it’s because they’re older and older but part of it’s because I’m older and older” (P08).
Decline in body flexibility	“Just in general getting stiff and harder to walk. I’ve got some spinal problems and that’s the decline in my body flexibility” (P13).
Changes in urinary function	“A little dribble now and then … and I find that annoying. It’s not so frequent that I’m really concerned or feel I need to start wearing whatever they call diaper or anything. It’s not that significant” (P07).
Changes in teeth or mouth	“They’re not that bad and I go to the dentist and he fixes them. So I haven’t changed my diet ... I still eat normal food. I still like to chew. I still eat raw broccoli, but teeth break, the fillings do not last forever” (P24).
Changes in digestion	“I have an issue with acid reflux ... it’s an ongoing issue, especially if I eat meat or spaghetti sauce or something. So that has come on in the last several years” (P22).
Changes in breathing	“When I go out for a walk, I may breathe a little bit harder” (P19).
Headaches or other pain	“The pain is ... like when the weather changes, then I have an issue with my back, my shoulders, my ankles. All my joints start acting up when the weather changes, in the wintertime specifically”(P19).
Changes in sleep	“I’m a much lighter sleeper than I used to be. I’ll wake up every hour and have to go to the bathroom. I have a hard time just having uninterrupted sleep” (P22).
Changes in sexual function	“I haven’t changed that much in that but my wife’s desires have changed. This is kind of a hard subject for me to address, but I think it’s harder for her too ... So to explain it in a short way, it doesn’t happen as much as I would like it to” (P22).
Cognition related	
Changes in memory	“Because of memory ... I think some of these are kind of the classics. It’s sometimes a little bit harder for me to find where I left my glasses because I can’t remember where I put them” (P08).
Easy to be distracted	“I can’t stay focused ... I’m learning how to live by myself, it’s like my brain goes off at a different pace” (P29).
Psychology related	
Psychological changes	“When I speak, I find that I will choke up. Maybe if I’m bragging about what a good job a grandchild did on something, and then I’ll just, it’s hard to finish the sentence, because I’m really feeling, ‘Wow! She did such a nice job,’ and the words aren’t coming out … why should I get choked up about a piano recital or something?” (P08).

The ARCs developed gradually: “It’s a very gradual change. I would say that it doesn’t come overnight. It takes a very long time. And I just noticed it as the years passed …” (P23).

Though the ARCs were related to the natural aging process, an ARC could also be a side effect of taking medications to treat another age-related disease. For example, a participant mentioned: “I take a diuretic (for atrial fibrillation), and that inclines one to have some of the symptoms (urinary symptoms) … that I would not have regularly” (P06).

### Response to the ARCs

Responses are the first reactions after participants realized their ARCs. Responses were categorized into emotional responses and behavioral responses. Many participants expressed surprise or negative emotions at the realization of the ARC: “I was saddened because I always thought my memory was so good” (P09), but then realized that these ARCs were part of aging and gradually accepted them: “It is surprising cause you just think you are young forever, and then all of a sudden I go, ‘Oh, well I am 70 something’. So that’s part of life” (P09).

Other participants expected these issues as they got older, so they graciously accepted the ARCs:

Well, I accept as someone who works in nature, I have a great acceptance for the fact that we are not made to last and that in nature, there’s a natural cycle of decline. As much as I would love to live forever, I know that that is not the natural cycle of nature. So I accepted (P23).

Some participants responded to their ARCs by starting to explore different adaptive strategies so that they could compensate for the capacity limitations and live a “normal” life: “My first response was to exercise more, make it harder, I need to do more, I need to go up steeper hills, I need to hike more” (P24).

### Adaptations

#### Adaptive behaviors

Different adaptative strategies emerged throughout the interviews. General adaptive strategies, like seeking healthcare, were executed to understand and find solutions to the ARC. “I have gone twice to audiologists. Just to make sure that there isn’t something else” (P08).

For physical ARCs (e.g., changes in muscle strength, changes in flexibility), participants practiced adaptations like conserving energy and increasing or maintaining stamina: “Things that make me a little bit tired or take my energy and strength, I just try to space them out and not do them all about the same time. That’s how I’ve been managing it” (P03).

Participants became more conscious about planning and executing actions in their daily life after having an ARC:

It doesn’t affect very much except for planning better when I do errands and when I leave the house I think planning, it takes more planning. I’m going out, I think, Okay. I’m going to be out now for 2.5 hours. I’ve got a plan for finding a bathroom (P23).

Another adaptation used by participants was adjusting the physical environment to make it better fit their changing capacity level. For example, a participant mentioned that due to diminished muscle strength some of the drawers in their cabinets were hard to pull. The participant went on to share: “I called the hardware store and asked them if there was something they could do to help. I found that there is a product I can buy to spray that makes them slide better” (P03).

Some participants expressed asking for help from family and friends as an adaptation strategy: “I ask for help more often than I would ever in the past. And then also even with the kids, I’ll call my daughter and just say, okay, it’s time for you to come (over)” (P01).

Many participants mentioned hoping to better adapt and address their ARCs by living a healthy lifestyle, such as improving exercise activities or taking supplements:

Keep doing exercises at home, keep doing my stretches, and I’ve added some more with the spine, and I got some information on seated yoga, and I’m aware of the changes, like how hard it is to get up off the floor. So in a lot of older women, they have hip replacements, and I don’t want to do that. I stretch my hips all the time, trying to keep them moving. I don’t want to get overweight because that’s another issue. So it’s keeping up with it, healthy body, good diet (P24).

I’m taking a supplement that was supposed to help with that I think things have been a little bit better ... (P22).

Tools or technologies were utilized by many participants to adapt to their changing capacities:

I really like the walking sticks. They also have enabled me to feel less worried about stumbling or being on uneven ground. I have had both of my knees replaced a long time ago. And the only thing I could say about the walking sticks is I should have started sooner. They’re very, very helpful. So using those helps my strength and the tools that are available help me open jars. Sometimes things are heavy for me to move ... I have a dolly and when I want to move my outdoor plants and indoors, I know how to use those tools. I just haven’t found it to be too limiting (P02).

In addition, several participants reported adjusting on their mental expectations as an adaptation strategy they use to. One participant said: “ you have to take care of yourself and be realistic about you are getting old, you can’t do the things as well as you used to, and slow down and enjoy being old” (P09). Willpower was also important for some participants to better adapt and accommodate their ARCs. Many participants practiced “self-talk” to ensure they practiced certain adaptations: “maybe the biggest one is to not be embarrassed to ask somebody to say something ... I just have to remind myself, do not just try to cover it up. Just ask” (P08). Some participants used a spiritual approach as an adaptation: “I hope to be able to do more as my hand gets better, back to normal, which I’m just praying that it will” (P03).

#### Motivations for adaptations

Participants developed adaptations due to different motivations. Some ARCs interfered with daily living and activities by causing physical discomfort, which directly led participants to develop adaptive strategies to accommodate or compensate for the functional limitations caused by the ARCs: “It’s just difficult to do other things. If my wrists or arms shoulders are really hurting and it’s difficult to sleep at night if my hip pain is very bad because I can’t get comfortable” (P18).

Some participants developed certain adaptations because they needed social interactions. A healthy social relationship was important for the participants, and they tried to maintain a comfortable level of social interactions by overcoming the limitations caused by the ARCs:

I’m surrounded by people. I like to be around people, and particularly children. And children are difficult with hearing aids, because they have a high pitch and they often don’t have very good enunciation. So the hearing is perhaps the highest in keeping me feeling that I participate in the world around me (P02).

Another strong motivation for participants to develop adaptations for the ARC was to have the adaptations not only accommodate their needs but also to prevent future limitations:

If I become less mobile, I might need some sort of assistive device. But if I can maintain like I am now I shouldn’t need it. I think that’s motivation for me to maintain like I am now or to keep going to add to what I’m doing now, so that doesn’t happen. I don’t want that to happen (P03).

In addition, others’ suggestions were also a factor that inspired participants to make adaptations:

I know when I first started having a hearing loss it was 15 years ago, and my oldest son told me I might need hearing aids. I didn’t agree with him. I found out later that he knew before I did (P02).

The sense of responsibilities for others also played an important role in motivating older adults to develop adaptations for their ARCs. For those who serve as caregivers for their loved ones, they needed to overcome their ARCs to perform their responsibilities:

I had the responsibility of that house and him (the husband) for 12 years. And I couldn’t let go of taking care of him, so I moved to reduce my drive to half an hour each way. And so, I moved here. After 12 years I ran out of steam. And I knew in order for me to be able to take care of him, I had to change my way of living (P17).

#### Barriers in making the adaptations

Participants discussed challenges they encountered while carrying out the adaptations, such as unwillingness to ask for help: “One of my biggest weaknesses is that I do not know how to ask for help ... It makes me even tear up thinking about asking for help. It’s something I’m just not comfortable. So, that will be very challenging for me” (P01).

The fact that other people might be unaware of or forget about the ARCs caused challenges for participants to perform their adaptations. Participants reported often having to remind others of their limitations:

I’ve always found the people around me cooperative, but I wouldn’t say that I got help from them, because they don’t recognize what I need. I’m the only one that seems to know ... My daughter, who’s very close to me and very accommodating, forgets sometimes and turns away from me, or talks to me from another room. Usually, I’m the one who has to ask, I have to remind her that I have to be in the same room ... I get help from people, but it’s usually my initiation rather than theirs (P02).

In addition, some participants mentioned that a lack of services was a barrier for them to carry out the adaptations, especially during COVID: “I go to classes ... I’ve joined gyms, but none of that’s available to me (because of COVID)” (P20).

Besides the previously mentioned barriers, some participants mentioned that they were reluctant to perform the planned adaptations because they did not like performing the adaptation or they are still considering implementing it:

I think it’s kind of hard because sometimes water just ... I know it’s good but it just tastes so bland. And a doctor told me that I needed to drink more And I’m trying to be better at that I would say I just need to hydrate more ... I just have to force myself to do it. I should probably go get water now (P22).

#### Attitudes toward the adaptations

Overall, participants were satisfied with their adaptations: “I’m pretty satisfied. If I weren’t, I would have done more ... I think the changes are good. I’m getting older, despite my efforts. But, I’m satisfied with what I’m doing” (P08). Most participants did not view their adaptive strategies as perfect because they are still not quite as capable as before even with the adaptations: “So it’s just that I do not have quite the stamina and I think you have your pride, when you think you’re the strongest people in the whole wide world” (P09). Besides, despite participants thinking their current strategies fulfilled their needs, they still perceived the need to be more diligent with the adaptations: “I just need to be more disciplined about putting liquid into my system. Just have to do it” (P22).

In addition, several participants mentioned that they are still trying to figure out the best solutions: “Well, I’m finding additional ways to help maintain my memory …” (P07). Moreover, some participants thought that there were factors that were out of their control:

Well, there’s some things I don’t have control over. I feel like I’ve done everything along the way, like got new hearing aids, better hearing aids, I ask people. But there’s still a percentage of things I can’t control about the hearing. I would like everybody to learn sign language. I think they should teach it in schools. I did work with deaf people and deaf kids for a while. But I don’t have control over that (P02).

Some participants mentioned that they are aware of other adaptations, which may help with their situation, but they have not yet practiced:

I know that there would be benefits if I did upper body exercises that were not labor. I know that I could look into that and I could do some of those exercises and I don’t really bother doing it (P23).

Most participants thought the strategies they employed worked well for their needs. Participants usually did not think their ARCs progressed to the level that requires additional effort to manage: “They’re the same (to manage) … right now, it’s not a big thing” (P19). Only a few of the participants thought the ARCs were more difficult to manage over time: “More difficult (to manage), slightly as time goes by ... I’ve asked about different things to do with my body and doctors have told me there’s not much you can do about it” (P16).

#### Result of adaptations

Performing adaptive strategies helped increase or maintain older adults’ independence by meeting their needs: “I’m not resistant at all to transition. I have no hesitation to just keep abreast of what will be helpful. I can’t understand why anybody would, because it will make me more independent” (P02). Meanwhile, participants stated that they were emotionally satisfied with their functional capacity and adaptive strategies: “So that seems to soothe me when I feel I’m frustrated. I just feel like, well, there’s an answer out there someplace I’ll find it. I do not feel hopeless very often, or helpless, either one” (P02). Notably, many participants expressed that they felt more confident as a result of performing the adaptive strategies: “The more I adapt, the more in control I am” (P15).

### Knowledge and Understanding of ARCs

Participants’ knowledge about their ARCs was from different sources. Many participants mentioned that their knowledge was from health care advice, or from observing peers or family members who have had a similar issue. Several participants were retired physicians or nurses and mentioned that their knowledge was from their career as health care providers. Another important source of knowledge was media, including but not limited to newspapers, online forums, and websites like the American Association of Retired Persons. Interestingly, many participants mentioned that their knowledge of the ARCs was accumulated based on their experience living with the ARCs over the years: “I guess just trial and error. Just trying different things” (P18).

Common features were observed among participants who had abundant knowledge of the ARCs. First, they tended to be precautious about the ARCs’ onset and progression: “I hope I’ll have sense enough to deal with it before it affects my ADLs (activities of daily living)” (P08). Second, they were more mentally prepared about the onset and progression of the ARCs, which led to better acceptance of the changes: “I knew it was coming. I realized that it’s part of the golden years …” (P19).

### Resources

Resources were important for participants to successfully develop and implement their adaptations. We summarized the resources that older adults utilized to assist with their adaptations in Table 3. *External resources* refer to tangible and intangible support received from other people. *Internal resources* refer to the unique, intangible personal features that equipped the participant with skills or capacities.

**Table 3.** Capacities and Resources

External resources	Quotes
Support from family members	“I’m lucky enough to have, my brother and sister-in-law, niece and nephew living next door …sometimes I have just big, hard things to do. And I ask them if they would participate … I say, ‘Do you have time to dig a deep hole to plant my new tree?’ I really can’t do that very easily anymore” (P23).
Support from friends	“And then at the orchard, I have availed myself to some of the younger people. When we have hard physical work to do, I ask them if they could do it. And I do some of the things that are more instructive rather than doing the job myself” (P23).
Help from professionals and/or hired help	“I do go to a chiropractor. I try to go to the chiropractor at least once a month. Then, I go get a massage ... Try to get a massage every so often, about every other month” (P19).
Support from community and faith organizations	“We have our church meets online … and I think that brings us a lot of comfort and calmness … Having a good church and a good bunch of folks that we know is good” (P20).
Insurance or money	“I do buy dental insurance on my own, it’s not part of Medicare. I’ve been paying for dental insurance for many years, and I believe it’s important. So I go to the dentist, I take care of my teeth” (P24).
Internal capacities	
Influences from childhood	“That’s how I was raised on the farm … there were all kinds of things that you did at certain stop times … my mother … she almost died ... So … I had to make meals for, do the laundry and all that sort of thing. So I got pretty savvy at what I needed to do …” (P09).
Influences from adulthood	“All of my adult life, a very curious person, a resourceful person. My work life was involved, reaching out and working with communities, and teaching, and being a resource person, so that’s part of who I am” (P29).
Physical capacity	“Here I am at 72 years old and I can do the things that I do which a lot of people younger than me, I do not think could handle much as I do. But like I said, I grew up working on the farm and being a nurse for as long as I was, I bet I still maintain a high quality of strength” (P09).
Emotional capacity	“I fight and then I lose and then I fight some more, but this is enough”(P17).
Cognitive capacity	“I learned with the work that I did, with my, paperwork, I learned how to function and still be comfortable with being distracted from a task. So I could leave a task and attend to whatever was happening, that needed to be taken care of at that moment, and then go back to the task” (P29).

## Discussion

This qualitative study applied a theory-driven approach to describe the experience of older adults adapting to their ARCs (i.e., ARCs and linkages to the challenges, adaptation strategies, and resources used). Participants identified and described their ARCs including the challenges encountered as well as strategies and resources used to address them. Examples were provided from all participants of how the strategies worked or did not work to address the ARC; acknowledgment of the friends, family, and providers who were consulted; and resources and tools that were developed, borrowed, or purchased.

The findings from this study substantiate the EWA framework. The sources of information about EWA were 2 years of weekly EWA blog posts written by Ms. Carnevali based on her nursing knowledge and aging experience (Carnevali, 2022) and five publications (Belza & Primomo, 2019; Carnevali et al., 2019; Ge, Belza et al. 2021; Primomo & Belza, 2019; Su et al., 2019). The current study, consistent with EWA, found that the majority of the participants addressed their ARCs and employed strategies that were effective to reduce the impact of the ARCs. The adaptations were individualized, showing creativity and effective problem-solving. Resources used included people (family, friends, neighbors, and professionals), the Internet, technology such as Zoom, and tools such as walking sticks. The motivations to make changes also varied and were largely based on how much the ARCs affected one’s daily living. Those who experienced successful adaptations were very proud and happily shared their approach. Barriers to making adaptations included unwillingness to ask for help, being unaware of their own limitations, lack of resources, and still being in the contemplative stage to making a change. The barriers identified in this study are consistent with what has been described in Carnevali’s blog (Carnevali, 2022) as well as consistent with previous literature on age-related changes and related behavioral changes (Bai et al., 2018; Whitelock & Ensaff, 2018; Wildenbos et al., 2018; Wilson et al., 2021). There were many outcomes of the adaptations made but most importantly, the adaptation allowed the participants to remain independent and confident in their approach.

When considering their adaptations to the ARCs, some participants’ behaviors aligned with the precontemplation and contemplation phases of the Transtheoretical (Stages of Change) Model (Glanz et al., 2015). The participants had tangible or intangible resources to initiate an adaptation, but their ARCs were not so disruptive that behavioral change was urgent. This relationship between adaptations to ARCs and the transtheoretical model was especially relevant for adaptations that involved increased physical activity and the acquisition of various forms of help from other people. The individual variations where participants fell in the transtheoretical model corresponded with their behaviors in the EWA phases of creating adaptive strategies and purposeful management of daily living (Primomo & Belza, 2019). Overall, participants proactively and purposefully implemented adaptations or planned future adaptations.

Participants described their experiences of recognition of the ARCs, their impact on daily life, and the strategies or resources used to navigate and achieve objectives (e.g., independence). Notably, participants discussed how they used their internal and external resources to effectively adapt to their selected ARCs. Individuals described that after the ARC’s onset, the corresponding adaptations evolved along with their changing ARCs. Key examples are some participants identified changes in muscle strength then adapted by incorporating regular physical activity in their routines. Others experienced decreased flexibility and became more open to requesting help from external resources such as friends and family. Our study also found that self-talk was a valuable practice that motivated individuals to manage their various ARCs. Self-talk and planning drove individuals to use their creativity and resources to facilitate satisfactory adaptations, which EWA blog posts also highlighted (Su et al., 2019).

This study was conducted during the global COVID-19 pandemic, which may have inhibited participants’ ability to adequately adapt to certain ARCs. Multiple participants with reduced hearing mentioned that mask mandates had restrained them from utilizing their adaptive strategy of reading speakers’ lips in face-to-face conversations. Multiple articles supported that COVID-19 mask mandates and social distancing were barriers for older adults with impaired hearing (Hulzen & Fabry, 2020; Knollman-Porter & Burshnic, 2020). Masks hindered the ability to read lips and facial expressions while social distancing, which reduced speech audibility (Hulzen & Fabry, 2020). Another COVID-related barrier to fulfilling ARC adaptations that multiple participants experienced was the closure of community centers and gyms. Closures of such facilities interfered with participants’ social activities and exercise routines. Our findings were consistent with other studies, which suggested that older adults were especially at risk for reduced physical, mental, and social well-being during COVID-19 because of limited access to community activities (Carriedo et al., 2020; Chen et al., 2021; Harden et al., 2020; Muller et al., 2021).

There were several limitations in this study. First, the generalizability of our qualitative findings is limited by the characteristics of our study sample, which were predominantly Caucasian women, residing in an urban area, and highly educated. Our sample had access to and were comfortable with using computers and the Internet. Additionally, most participants either already knew how to access Zoom or were willing to learn. The sample had access to health care providers, which likely provided them with more options in medically managing ARCs. Not represented in our sample were those with low computer literacy and less access to health care. Heritage, culture, and social–economic status influence one’s reaction to and ability to address age-related changes. Although we know the analysis of qualitative data is less concerned with generalizability (Polit & Beck, 2010), the characteristics of the sample may have influenced what we found. A future study might consider including a more diverse sample in regard to education, ethnicity, and computer literacy to determine whether their experience with ARCs is similar to or different from this sample. However, given that this current study is qualitative rather than quantitative, the goal is to provide a rich description, not an exhaustive description of the experiences of all older adults (Polit & Beck, 2010). A second limitation was that participants may have responded based on what they thought was a socially acceptable response. To address this potential bias, we formatted questions to be open-ended and told participants there was not a right or wrong answer. We did not use leading questions that might prompt the participant to respond in favor of a particular response. A third limitation was that we did not systematically collect information on participants’ health conditions other than what participants self-disclosed during the interviews. We believe this limitation does not weaken this study as it primarily focuses on older adults’ self-perceived health challenges related to aging and their adaptive behaviors. However, future studies of EWA might consider collecting information on health conditions in order to gain a more holistic understanding of participants’ situations.

Our findings have important clinical and research implications. In health care settings, providers and caregivers could include the status of and adaptation to ARCs during healthcare visits. The information can then be used to develop individual care plans and tailored patient-centered interventions with them. In community settings such as senior centers or retirement residences, caregivers, or staff could consider using ARCs information (e.g., the individual adaptation to ARCs and search of resources) to arrange the most suitable activities accordingly. We envision that future studies can utilize the EWA framework and proposed ARCs to promote the development of individual care plans and tailoring person-centered care for the aging population. Future research could include participants with low computer literacy and a range of education levels, as well as greater ethnic diversity. Future studies should also consider focusing on the ARCs among older adults with frailty and those who reside in nursing homes.

A notable, innovative highlight of the EWA framework is its emphasis on individuals’ capacities to effectively manage ARCs. This strength and flexibility-based approach is a more positive lens through which to study aging compared to deficit-based analyses of aging. Given the trend of global aging, it would be helpful to have students in nursing and allied health care academic programs learn how to serve aging adults by bolstering their capacities rather than narrowly examining their emerging deficits. EWA concepts are, therefore, necessary additions to academic curricula. The integration of the EWA framework into a course at a college or university is worthy of consideration. Physicians, nurse practitioners, physical therapists, and occupational therapists among many other clinicians are integral to the holistic care of their aging patients. The EWA framework is essential to these medical professionals’ conceptualizations of how individuals can age well, learn to adapt to their ARC’s using their own capacities, and live an optimal quality of life amidst the physical and psychosocial trials that accompany aging.

## Conclusion

This qualitative study and in-depth analysis of older adults’ ARCs and adaptations demonstrated that older adults developed individualized adaptations to fit their needs and capacities. The findings contribute to the best of our knowledge of aging by providing a rich description of the experiences of ARCs and associated adaptations. Findings from this study provide evidence to support EWA by showcasing its applicability among older adults. EWA provides a lens to study the individual aging experience, which will continue to contribute to our understanding and development of person-centered care.

## Supplementary Material

igac054_suppl_Supplementary_Material
