# Supplementary material for: Engaging With Aging: A Qualitative Study of Age-Related Changes and Adaptations
Source: Innov Aging. 2022 Oct 11;6(6):igac054. doi: 10.1093/geroni/igac054 (PMC9701056; doi:10.1093/geroni/igac054)
Supplement: igac054_suppl_Supplementary_Material [file igac054_suppl_supplementary_material.docx]

Online Supplementary Material: Interview Guide

I have on the screen a white board with post-its. Can you see the posts-its? The post-it notes contain common age-related changes in capacities. We will go through each post it and move each one into one of two categories. If it is a change that you have experienced as a result of getting older, we will put it under “yes” If it is a change you have not experienced as you get older, we will move it under “no”. In the end, you may find that you have experienced some changes that are not listed among the post-it notes. We can add extra post-it notes to your experienced pile.

*[Move Jamboard post-its based on discussion with participant. Create new ones as needed]*

Now, we will deal only with the cards in your experienced category.

Some of the age-related changes may have reached the point where they interfere with your daily living. You may find that you have had to make adaptations in order to live the way you want to. Other changes may not have reached that point yet.

As you go through the “experienced” post-it notes, we will put those that have caused you to make adaptations in the way you do things, into a pile on the left side. We will move those that have not required you to make adaptations on the right side.

{Pause}

Now, let us look at the items that caused you to make adaptations in the way you carry out your daily living. Please choose three that have caused the greatest challenges on your life. Please rank them from the greatest challenge to the least. [*Interviewer should move the Post-it notes around so they are in rank order or mark the numbers on top*.]

I will ask a set of questions for each of these age-related changes. When answering the questions, please focus on one specific age-related change at a time. I will ask you about other age-related changes that you have experienced later.

1. Tell me more about XXX. *[Let the participant answer before asking the probes]*

Probes: *[Ask the ones that haven’t been covered in participants’ answer]*:

What XXX is like for you? *[Aim to seek for details, e.g., people may mean different things by saying “changes in flexibility”. Is that hip? Is that overall? Is that knees?]*

Please tell me your experience with XXX.

What activities have become harder because of your changed capacity?

Can you give me an example?

2. Let’s talk about the ways you have dealt with XXX.

a. How have you dealt with XXX?

Probes:

What have you done to manage XXX? What are you doing differently?

b. How did you figure out how to manage XXX? [aims to seek for information in how have participants figured out the adaptations]

c. In what ways have you involved others (family, friends, neighbors, healthcare providers) in managing XXX?

Probes:

What type of help did you ask for from others (family, friends, neighbors, healthcare providers) in planning for ways to deal with this change?

What type of help did you ask for from others (family, friends, neighbors, healthcare providers) when carrying out your adaptations?

d. On a scale of 1 - 10 how satisfied are you with the way you have managed the age-related change? Where 1 is not satisfied and 10 is very satisfied. Why did you pick #?

e. As time has gone by, does it seem to you that your abilities to manage XXX have become easier, more difficult or stayed the same? . Please explain.

Probe:

In what ways, if any, has the COVID pandemic change your ability to manage or deal with the age related change

3. Tell me more about your response when you first noticed XXX.

a. When you first noticed XXX, how did you feel about XXX? How do you feel about it now?

Probes:

When did you first notice this change?

Thinking back to when you first noticed this change, what caused you to notice it? *(for Hillary!)*

Which activities in your daily life were the first to be affected? *(for Hillary!)*

b. Before you had XXX, did you know about XXX? If yes, did you know what might have caused XXX?

c. After having it, did you learn more about XXX? If yes, through what ways?

4. Tell me about your plan for the future related to XXX.

a. XXX may progress, are you planning ahead more adaptations for future changes? Yes No Not sure. Please explain.

b. What tasks of daily living do you anticipate future changes in XXX may affect?

5. Is there anything else you would like to say about how you adapted to XXX?

6. Overall, do you feel the ways you are adapting to the changes of your aging gives you more, less, or the same amount of control? Please explain.

----------------------------------------------------------------------------------------------------------------

Now let’s look at the post it notes where age-related changes are occurring but don’t require you to make any adaptations in the way you do things at this time. Please choose three that have caused relatively greater impact on your life.

7. Why do you think [this age-related change] does not require you to make any lifestyle changes?

Probes:

Any activities have become harder because of your changed capacity?

8. Tell me more about your response when you noticed XXX.

a. When you first noticed XXX, how did you feel about XXX? How do you feel about it now?

Probe: When did you first notice this change?

b. Before you had XXX, did you know about XXX? If yes, did you know what might have caused XXX?

c. After having it did you learn more about XXX?

9. Has XXX stayed the same, gotten worse or gotten better since you first noticed it??

Probe:

What tasks or areas of daily living do you anticipate it may affect when it progresses?

10. Is there anything more you would like to share about your experience with aging?

This completes the interview.
